# Optimisation of quantitative miRNA panels to consolidate the diagnostic surveillance of HBV-related hepatocellular carcinoma

**DOI:** 10.1371/journal.pone.0196081

**Published:** 2018-04-19

**Authors:** Ngo Tat Trung, Dang Chieu Duong, Hoang Van Tong, Tran Thi Thu Hien, Phan Quoc Hoan, Mai Hong Bang, Mai Thanh Binh, Thai Doan Ky, Nguyen Lam Tung, Nguyen Tien Thinh, Vu Viet Sang, Le Thi Phuong Thao, C-Thomas Bock, Thirumalaisamy P. Velavan, Christian G. Meyer, Le Huu Song, Nguyen Linh Toan

**Affiliations:** 1 Department of Molecular Biology, 108 Military Central Hospital, Hanoi, Vietnam; 2 Department of Gastroenterology, 108 Military Central Hospital, Hanoi, Vietnam; 3 Vietnamese-German Center of Excellence in Medical Research, Hanoi, Vietnam; 4 Department of Pathophysiology, Vietnam Military Medical University, Hanoi, Vietnam; 5 Viet Tiep Hospital, Le Chan District, Hai Phong, Vietnam; 6 Institute of Biomedicine and Pharmacy, Vietnam Military Medical University, Hanoi, Vietnam; 7 Institute of Tropical Medicine, University of Tübingen, Tübingen, Germany; 8 Institute of Clinical Infectious Diseases, 108 Military Central Hospital, Hanoi, Vietnam; 9 Department of Pharmacy, 108 Military Central Hospital, Hanoi, Vietnam; 10 Department of Infectious Diseases, Robert Koch Institute, Berlin, Germany; 11 Duy Tan University, Dan Nang, Vietnam; Centre de Recherche en Cancerologie de Lyon, FRANCE

## Abstract

**Background:**

Circulating microRNAs (miRNA) are biomarkers for several neoplastic diseases, including hepatocellular carcinoma (HCC). We performed a literature search, followed by experimental screening and validation in order to establish a miRNA panel in combination with the assessment of alpha-fetoprotein (AFP) levels and to evaluate its performance in HCC diagnostics.

**Methods:**

Expression of miRNAs was quantified by quantitative PCR (qPCR) in 406 serum samples from 118 Vietnamese patients with hepatitis B (HBV)-related HCC, 69 patients with HBV-related liver cirrhosis (LC), 100 chronic hepatitis B (CHB) patients and 119 healthy controls (HC).

**Results:**

Three miRNAs (mir-21, mir-122, mir-192) were expressed differentially among the studied subgroups and positively correlated with AFP levels. The individual miRNAs mir-21, mir-122, mir192 or the triplex miRNA panel showed high diagnostic accuracy for HCC (HCC vs. CHB, AUC = 0.906; HCC vs. CHB+LC, AUC = 0.81; HCC vs. CHB+LC+HC, AUC = 0.854). When AFP levels were ≤20ng/ml, the triplex miRNA panel still was accurate in distinguishing HCC from the other conditions (CHB, AUC = 0.922; CHB+LC, AUC = 0.836; CHB+LC+HC, AUC = 0.862). When AFP levels were used in combination with the triplex miRNA panel, the diagnostic performance was significantly improved in discriminating HCC from the other groups (LC, AUC = 0.887; CHB, AUC = 0.948; CHB+LC, AUC = 0.887).

**Conclusions:**

The three miRNAs mir-21, mir-122, mir-192, together with AFP, are biomarkers that may be applied to improve diagnostics of HCC in HBV patients, especially in HBV-related LC patients with normal AFP levels or HCC patients with small tumor sizes.

## Introduction

Hepatocellular carcinoma (HCC) is the fifth and seventh most common cancer in males and females, respectively, and accounts for at least 700,000 deaths worldwide annually [1]. HCC patients at early stages or patients without concomitant liver cirrhosis (LC) have a favorable chance of curative surgery. The overall survival time of HCC patients undergoing timely surgery can reach five years [1, 2]. However, the 5-year gross survival for all HCC patients is lower than 10% [3]. The high mortality of HCC is due to the lack of suitable tools for early detection, as well as an unfavourable response to chemotherapy [4]. Chronic hepatitis B virus (CHB) infection is the leading cause of hepatic cirrhosis, which is the major risk for HCC. Therefore, the implementation of effective and reliable strategies to screen hepatitis B virus (HBV) carriers and to detect HCC at early stages will improve survival rates. So far, ultrasound and the assessment of serum AFP/DCP/AFP-L3 (alpha-fetoprotein/Des-gamma-carboxy prothrombin/lectin reactive AFP [isoform of alpha-fetoprotein]), is recommended for surveillance and early screening of HCC in high-risk groups [5, 6]. However, the interpretation of imaging data depends on tumour sizes and diagnostic skills and, thus, may be difficult in patients whose tumour develops on the background of other conditions such as obesity, cirrhosis, benign liver hemangioma and parasitoses such as liver fluke and other parasitic infections. Furthermore, a meta-analysis has indicated that the sensitivity of hepatic ultrasound is only 63% in the detection of early-stage HCC, as defined according to the Milan criteria (one nodule <5 cm or three nodules each <3 cm in diameter without gross vascular invasion) [7]. In addition, the performance of currently used serum protein biomarkers for routine surveillance of HCC is unsatisfactory. Assessment of serum AFP levels at a cut-off of 20 ng/mL has a sensitivity of 25–65% in the diagnosis of HCC and only 14–40% for pre-clinical disease [6, 8, 9]. Sensitivity and specificity of DCP in the diagnosis of HCC are 28–89% and 87–96%, respectively, values similar to those of AFP-L3 [10]. Therefore, new biomarkers with higher accuracy and with the ability to complement hepatic imaging are needed to improve the diagnostics of HCC.

MicroRNAs (miRNA) are a class of small and endogenous non-coding RNA molecules known to post-transcriptionally modulate gene expression by negatively regulating the stability or translational efficiency of their target mRNAs. miRNAs are involved in controlling a wide array of biological processes in cell proliferation, differentiation and apoptosis [11]. Aberrant expression of miRNAs has been widely reported in various types of human diseases, including malignancies [11, 12]. Several studies have aimed to establish panels of circulating miRNAs for early detection of HCC. However, the proposed panels appear to be specific for distinct ethnic groups [13, 14], and their serum expression was so far mainly compared between HCC and non-HCC groups. The challenge in HCC surveillance is to decipher the overlap between the early stages of HCC and LC, as well as to discriminate early stage HCC patients void of LC from chronic HBV carriers. Moreover, there is no consensus in terms of individual miRNA molecules used in the previously reported panels [14–16]. Importantly, the use of the proposed miRNA panels in combination with serological AFP levels in HCC diagnostics has so far not been evaluated comprehensively.

We performed a literature search, selected potential miRNAs and screened and validated those miRNAs in a cohort of HBV patients with different stages of liver disease progression. We established a miRNA panel in combination with the measurement of AFP levels and evaluated its diagnostic performance in surveillance of HCC.

## Materials and methods

### Search strategy and selection criteria for miRNA

We systematically searched the PubMed, BioMed Central, and Science Direct databases for studies that assessed individual miRNAs or miRNA panels in the differential diagnosis of HBV-related liver diseases. Search terms used in various combinations were “serum”, “plasma”, “microRNA”, “miRNA”, “HCC”, “liver cancer”, “hepatocellular carcinoma”, “liver cirrhosis”, “diagnostics” “diagnosis”, “detection” and “HBV-related HCC”. The studies were restricted to those published in English. Only miRNA molecules described in studies with more than 100 patients in each subgroup (HCC, liver cirrhosis, chronic HBV infection and healthy controls) and expression of selected miRNAs changes at least two times between studied groups were considered for further analyses.

### Study design, screening and validation

**Fig 1:**
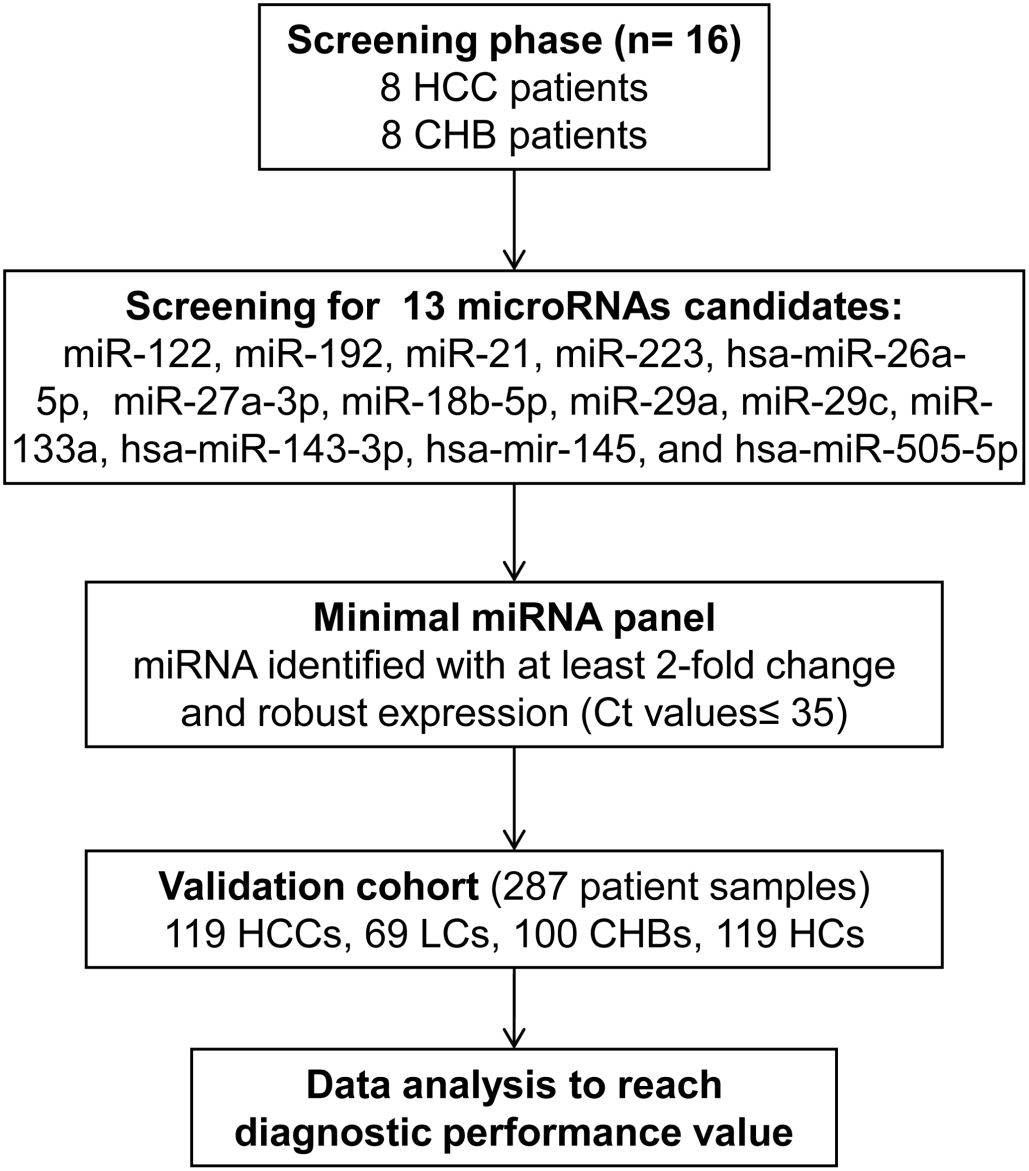
Study design. miRNAs described in studies with more than 100 HCC patients were considered for screening in eight HCC and eight CHB serum samples. Only miRNAs differentially expressed at least twice were selected for further validation.

Based on our search strategy and selection criteria, the miRNAs were quantitatively screened in 16 serum samples from eight HCC and eight CHB patients (matched for age and gender, Fig 1). Only miRNAs that differentially changed their expression level of at least two times between HCC and CHB groups were selected for further evaluation. For robust screening, only miRNAs with cycle threshold (Ct) values lower than 35 were considered to be expressed in clinical samples. The parameters of the logistic regression model from the screening phase were applied to a cohort of 287 samples for validating the diagnostic performance of the selected miRNA panel.

### Patients and sampling

Serum samples from 287 Vietnamese patients with HBV-related liver diseases were collected between 2014 and 2016. The patients were categorised into subgroups based on clinical symptoms, biochemical and liver function tests as well as the etiology of the liver disease [17]. The patient groups included 118 patients with HBV-related hepatocellular carcinoma (HCC), 69 patients with HBV-related liver cirrhosis (LC) and 100 chronic hepatitis B virus carriers (CHB). We also included 119 healthy individuals as a control group (HC). All HCs were confirmed by Elisa tests to be negative for HBsAg, HCV Ab (Dia.Pro Diagnostic Bioprobes Srl, Milan, Italy), HIV Ag/Ab combination (Murex Biotech Limited, Temple Hill, UK). In addition, the HCs were negative for HCV/HBV/HIV nucleic acid as assessed by HCV/HBV/HIV Real-TM Realtime PCR kit (Sacace Biotechnologies Srl, Como, Italy).

Biopsies were taken from all patients with suspected chronic HBV infection and, based on histological examination, classified into groups with or without evidence of either LC or HCC, In the case of HCC, the degree of differentiation was determined. All individuals of the HCC group presented a late stage of carcinoma. Individuals with neither LC nor HCC were attributed a histological activity index according to the scheme described previously [18]. Blood samples were obtained from all patients and HCs and serum was immediately separated and stored at -70 °C until further use.

The study was approved by the institutional review board and an Independent Ethics Committee of the 108 Military Central Hospital, Hanoi, Vietnam. Informed written consent was obtained from all study patients.”

### Biochemical and serological tests

The levels of albumin, globulin, total and direct bilirubin, alanine transaminase (ALT), aspartate aminotransferase (AST) were measured on an auto-analyser (Hitachi Automatic Analyser, Tokyo, Japan). ALT and AST were assessed for both the patietns and HCs. Markers for HBV infection (HBsAg, anti-HBc-IgM, anti-HBcIgG, HBeAg, anti-HBe) were assessed by commercial immunoassay kits (General Biologicals Corp, Taipei, Taiwan and DiaSorin, Saluggia, Italy). AFP was measured using a commercial ELISA kit (General Biologicals Corp., Taipei, Taiwan).

### miRNA extraction and cDNA synthesis

Total RNA, including miRNA fractions, was isolated from 200 μl serum with TRIzol reagent and reconstituted in 50 μl water treated with diethylpyrocarbonate (DEPC). The quality of total RNA preparations was assessed by NanoDrop spectrometer (NanoDrop Technologies, Wilmington, USA) at 260 and 280 nm (A260/280). Approximately 300 ng of total RNA were used for reverse transcription (RT) by RevertAid First Strand cDNA Synthesis Kit (ThermoFisher Scientific Inc, Singapore) following the manufacturer’s instruction. Primers used for cDNA synthesis were designed according to stem-loop theory as described previously [19]. Primer sequences are provided in the S1 Table.

### Quantification of miRNA by real-time PCR

After reverse transcription, cDNA was reconstituted in 100 μl 25 mM-Tris-HCl pH 8.0. The real-time PCR (qPCR) reaction mixtures consisted of 10 μl of 2x Sybr-Green I master mix (Applied Biosystems, Foster City, CA, USA), 5 μl of cDNA preparation, 5 pmol of miRNA universal reverse primer GTGCAGGGTCCGAGGT and 5 pmol of forward primer specific for miRNA. (S1 Table). The qPCR reaction was performed using the Stratagene M3000p device (Stratagene, San Diego, CA, USA) with a pre-incubation step at 50 °C for 15 minutes, initial denaturation at 95 °C for 5 minutes, followed by 45 cycles of 95 °C for 15 sec and 60 °C for 60 seconds. The RT-PCR reactions were finalised by amplicon melting dissociation. The cycle of threshold (Ct) values were recorded and analysed according to the comparative Ct method [20], in which the Ct value of miRNA-16 was used as normalisation factor as recommended previously [21].

### Statistical analysis

Statistical analyses were performed by the SPSS software version 19. Values were presented as either mean with standard deviation (SD), median with 25–75% percentiles, or numbers with percentages where appropriate. The non-parametric Mann-Whitney U-test or Kruskal-Wallis test was used for pair-wise comparisons. Univariate analyses were conducted to determine the relationship between the expression level of each miRNA and the presence/absence of HCC (HCC vs. LC, CHB, and HC). Spearman’s rank correlation coefficient was used to test correlations between two variables. The diagnostic accuracy of miRNAs was evaluated by receiver operating characteristic (ROC) analysis and the area under the curve (AUC) was calculated. The predicted probability of a HCC diagnosis was used as a surrogate marker to construct ROC curves. AUC was used as an accuracy index for evaluating the diagnostic performance of the selected miRNA panel. Binary logistic regression analysis was done to evaluate the diagnostic performance of the selected miRNA panel. *P* values were two-sided and the level of significance was set at a *P* value < 0.05.

## Results

### Clinical characteristics of the patients

The clinical and demographic characteristics (age, gender, liver biochemical tests, viral loads, AFP levels) for the 287 Vietnamese HBV-positive patients as well as age, gender, AST, ALT and total and direct bilirubin of the 119 HCs are summarised in Table 1. White blood cell, red blood cell and platelet counts were increased in the LC group compared to the CHB and HCC groups (*P*<0.05). The levels of HBV loads, ALT, AST, total and direct bilirubin were significantly higher among CHB and LC compared to HCC patients (*P*<0.05). The levels of total protein and prothrombin were significantly decreased in LC compared to the CHB and HCC groups (*P*<0.05). As expected, the AFP levels were significantly higher in HCC patients compared to CHB and LC patients (*P*<0.05) (Table 1).

**Table 1:** Characteristics of study paticipants according to clinical presentation.

Characteristics	HC (n = 119)	CHB (n = 100)	LC (n = 69)	HCC (n = 118)
Age (years)	23.76 [19–75]	42.6 [19–85]	58.6 [27–87]	55.6 [23–92]
Gender (M/F)	92/27	92/8	52/17	108/10
AST	31.7 [20–37]	376.1 [26–6206]	128.9 [24–1038]	92.2 [20–563]
ALT	31.6 [21–36]	406.9 [9–3390]	86.4 [6–1000]	61.3 [11–391]
Total bilirubin (mg/dl)	8.8 [4.8–15.3]	55.1 [8.4–551]	70.4 [6.2–514.5]	19.0 [4.8–86.3]
Direct bilirubin (mg/dl)	4.7 [1.7–9.3]	25.8 [1.0–349]	31.8 [1.6–258.6]	7.1 [0.7–53.3]
Albumin	NA	39.4 [25.0–48]	31.3 [19–46]	38.5 [22.4–48]
Total Protein	NA	73.6 [51.0–90]	71.4 [54–97]	76.9 [59–92.6]
AFP	NA	43.9 [0.87–917]	26.6 [0.9–154.2]	565.1 [0.8–16660]
AFP < 20 ng/ml	NA	73 (73.0)	46 (66.7)	41 (34.7)
Prothrombin* (% of standard)	NA	87.5 [17.0–152]	60.0 [26.9–138]	93 [49–123]
WBC* 10^9^/L	NA	6.9 [2.9–16.2]	5.8 [1.7–15.2]	7.4 [2.89–17.9]
RBC* 10^12^/L	NA	4.8 [3.1–6.]	3.8 [2.2–6]	4.7 [3.1–7.2]
PLT* 10^9^/L	NA	186.6 [55–416]	90.49 [29–279]	191.8 [38–479]
HBV load* (copies/ml)	negative	2.3x10^8^ [<100–3.69x10^9^]	8.14x10^7^ [<100–1.25x10^9^]	3.11x10^6^ [<100–3.72x10^7^]
**Child-Pugh**				
A	NA	NA	29 (42.1)	105 (89)
B	NA	NA	25 (36.2)	4 (3.4)
C	NA	NA	15 (21.7)	1 (0.8)
Unknow	NA	NA		8 (6.8)
**Tumor number**				
1	NA	NA	NA	69 (58.5)
2–3	NA	NA	NA	16 (13.6)
> 3	NA	NA	NA	32 (27.1)
Unknown	NA	NA	NA	1 (0.8)
**Tumor size (cm)**				
< 2	NA	NA	NA	1 (0.8)
2 - < 3	NA	NA	NA	7 (5.9)
3 - < 5	NA	NA	NA	27 (22.9)
5 - < 10	NA	NA	NA	47 (39.8)
> 10	NA	NA	NA	35 (29.8)
Unknown	NA	NA	NA	1 (0.8)
**Maximum tumor size** (cm)	NA	NA	NA	7.7 [1.1–16.2]
**Vascular invasion**				
Yes	NA	NA	NA	30 (25.5)
No	NA	NA	NA	87 (73.7)
Unknown	NA	NA	NA	1 (0.8)
**Metastasis**				
Yes	NA	NA	NA	21 (17.8)
No	NA	NA	NA	95 (80.5)
Unknown	NA	NA	NA	2 (1.7)
**Tumor differentiation**				
High	NA	NA	NA	28 (23.7)
Intermediate	NA	NA	NA	53 (44.9)
Low	NA	NA	NA	11 (9.3)
Unknown	NA	NA	NA	26 (22.1)
**BCLC staging**				
O	NA	NA	NA	0 (0)
A	NA	NA	NA	5 (4.2)
B	NA	NA	NA	67 (56.8)
C	NA	NA	NA	36 (30.5)
D	NA	NA	NA	0 (0)
Unknown	NA	NA	NA	10 (8.5)

CHB, chronic hepatitis B; LC, liver cirrhosis; HCC, hepatocellular carcinoma; WBC, white blood cell; RBC, red blood cell; PLT, platelet; AST and ALT, aspartate and alanine amino transferase; IU, international units; AFP, alpha-fetoprotein; BCLC, Barcelona Clinic Liver Cancer stage; NA, not available. Values given are with medians and ranges;

*, *P* < 0.05 for comparison with all other groups.

### Screening results of selected miRNAs in HCC and CHB patients

According to our search strategy and inclusion criteria, 13 miRNAs (miR-122, miR-192, miR-21, miR-223, hsa-miR-26a-5p, miR-27a-3p, miR-18b-5p, miR-29a, miR-29c, miR-133a, hsa-miR-143-3p, hsa-miR-145, hsa-miR-505-5p) were selected for expression screening in serum samples of eight HCC and eight CHB patients. Expression of eight miRNAs (miR-21, miR-29a, miR-29c, miR-122, miR-133a, miR-143-3p, miR-192 and miR-223) were at least two-fold higher in sera of HCC compared to those of CHB patients (S1 Fig and S2 Table). During the subsequent experiments, we realised that it was virtually impossible to distinguish the weak RT-PCR fluorescent signals from artefacts. Therefore, only miRNAs with Ct values <35 were included in further analyses. miR-29a, miR-29c and miR-133a were excluded due to Ct values >35. The three miRNAs miR-21, miR-122 and miR-192 were significantly over-expressed in sera of HCC patients compared to CHB patients and met all selection criteria. Therefore, miRNAs miR-21, miR-122 and miR-192 were included for further validation (S2 Table).

### Expression of miR-21, miR-122 and miR-192 in HBV patients and healthy controls

**Fig 2:**
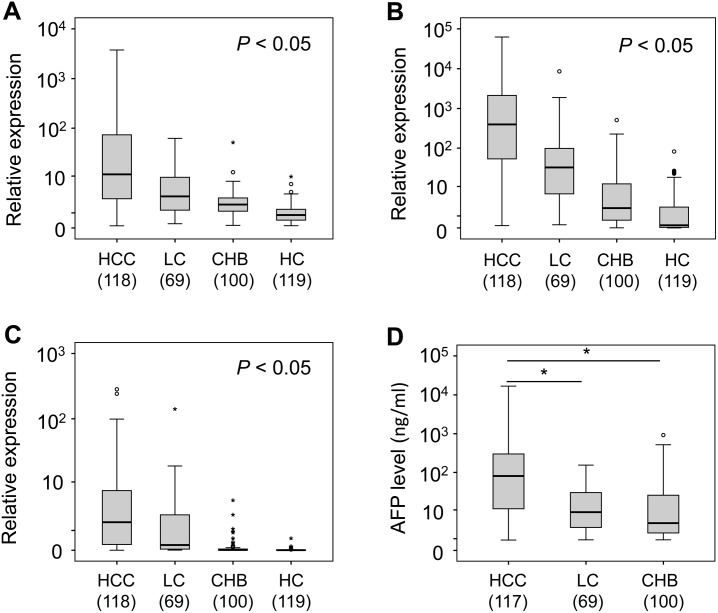
Differential expression miRNAs and AFP levels in different groups. Differential expression of miR-21 (A), miR-122 (B) and miR-192 (C) and alpha-fetoprotein (D) in different groups of HBV-related liver diseases including hepatocellular carcinoma (HCC), liver cirrhosis (LC), chronic hepatitis B (CHB) and healthy controls (HC). Numbers in brackets = n of individuals tested. *P* values were calculated by non-parametric Mann-Whitney U-test for pair-wise comparisons between groups.

In the validation phase, the parameters of the logistic regression model from the screening phase were applied to the diagnostic performance of the selected miRNA panel in the serum samples from the 287 HBV patients and 119 HCs. The results showed that the expression levels of miR-21, miR-122 and miR-192 differed between groups (*P*<0.05). Expression levels were highest in HBV-related HCC patients, followed by the HBV-related LC and CHB groups, and were lowest in HCs (Fig 2).

### Correlation between miR-21, miR-122 and miR-192 expression with age and clinical parameters

We analysed the correlation of circulating miR-21, miR-122 and miR-192 levels with clinical parameters in all 287 HBV-positive patients. Levels of miR-21, miR-122 and miR-192 were positively associated with AFP levels (Spearman’s rho = 0.21, 0.27 and 0.24, respectively, *P*<0.0001) (S2 Fig). However, when analysing the correlation of miR-21, miR-122 and miR-192 with AFP levels in the subgroups of CHB, LC and HCC, the associations did not reach statistical significance. There was no correlation between the expression levels of miR-21, miR-122 or miR-192 with age (S4 Table) and other clinical parameters.

### Expression of miR-192 and miR-122 is elevated in CHB patients with higher ALT levels

AST and ALT are indicators of liver damage. Normal ALT level were 20–40 IU/ml [22] in our groups. We classified 100 CHB patients into two groups based on AST and ALT levels (≤40 and >40 IU/ml) and compared the levels of miR-21, miR-122 and miR-192 between groups. miR-122 and miR-192 levels were significantly elevated in CHB patients with ALT levels >40 IU/ml (p<0.05) (S3 Fig), whereas miR-21 levels did not differ between the two CHB patient groups. In addition, no correlation between the miRNAs with serum AST level was observed.

### Association of serum miR-21, miR-122 and miR-192 levels with HCC stage

Prognosis and indication of any of the various treatment options HCC patients are dictated not only by tumour staging, but also by the degree of liver function impairment. Therefore, accurate assessment and classification of HCC is essential for patient management. The Barcelona Clinic Liver Cancer (BCLC) algorithm is a common staging system for HCC classification. BCLC relies not only on tumour and nodule size, but also on the degree of vascular invasion or extrahepatic spread and the Child-Pugh classification [23, 24]. We compared serum miR-21, miR-122 and miR-192 levels among HCC patients in different BCLC stages. We observed that the serum miR-192 levels were highest among HCC patients in BCLC stage A, followed by the HCC patient groups with BCLC stages B and C (*P*<0.05). The serum levels of miR-21 and miR-122 were lower in HCC patients with BCLC stage C compared to those with stage BCLC-B, however, the difference did not reach statistical significance (S3 Table). In addition, we compared the miRNAs levels among HCC patients with different tumor sizes and found that, when tumor sizes were >3 cm, the levels of miR-21, miR-122 and miR-192 were decreased with regard to tumor size. In contrast, AFP levels were increased when the tumor size was smaller than 10 cm. However, the difference did also not reach statistical significance (S4 Fig).

### Diagnostic performance of miRNA panels and AFP levels in differentiating HCC

**Fig 3:**
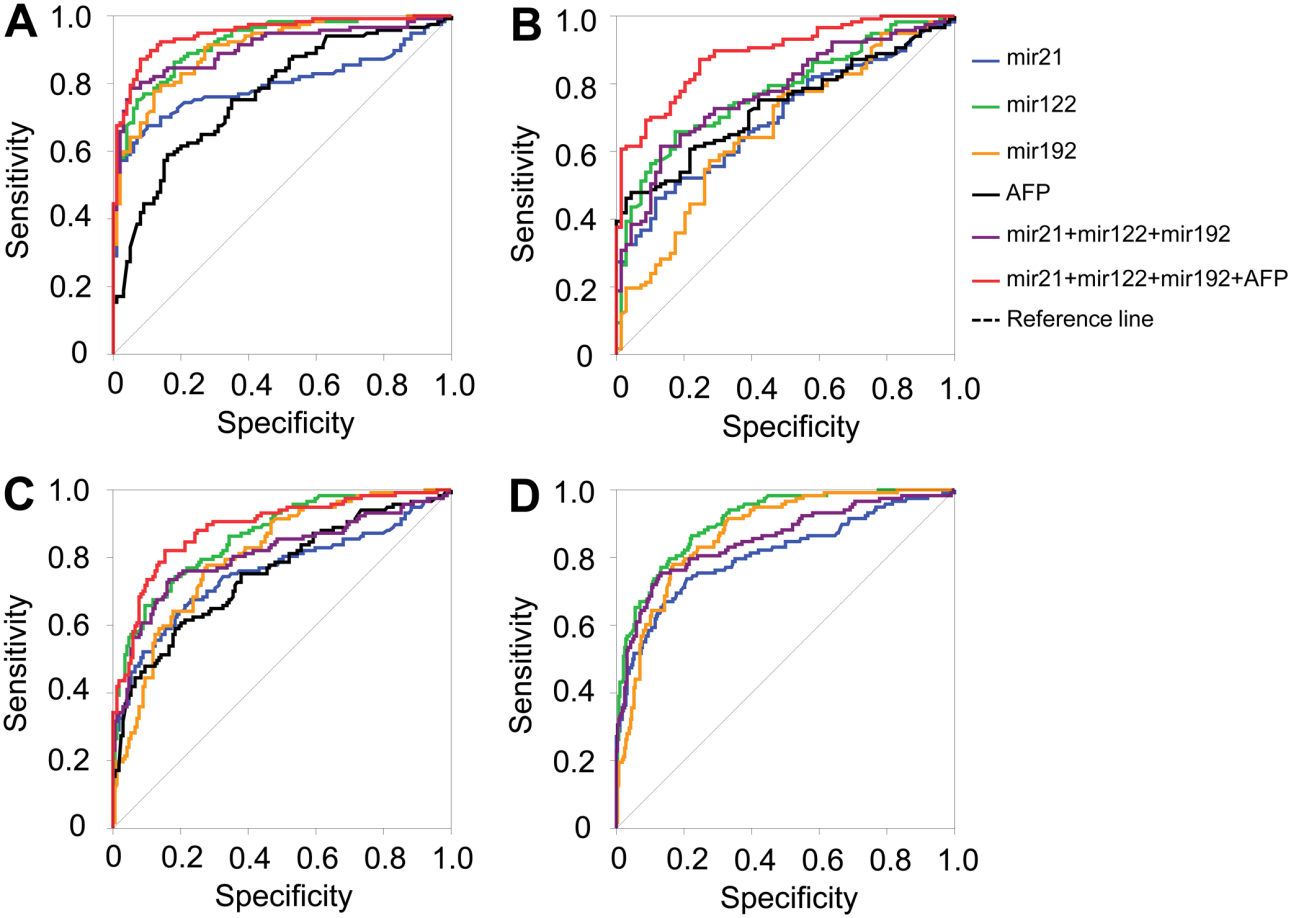
Diagnostics performance of miRNA panels and in combination with AFP levels. Diagnostic performance of the triplex miRNA panel based on the miRNAs miR-21, miR-122 and miR-192 levels in differentiating hepatocellular carcinoma from other conditions. (A): HCC vs. CHB; (B): HCC vs. LC; (C): HCC vs. LC+CHB and (D): HCC vs. (LC+CHB+HC).

**Fig 4:**
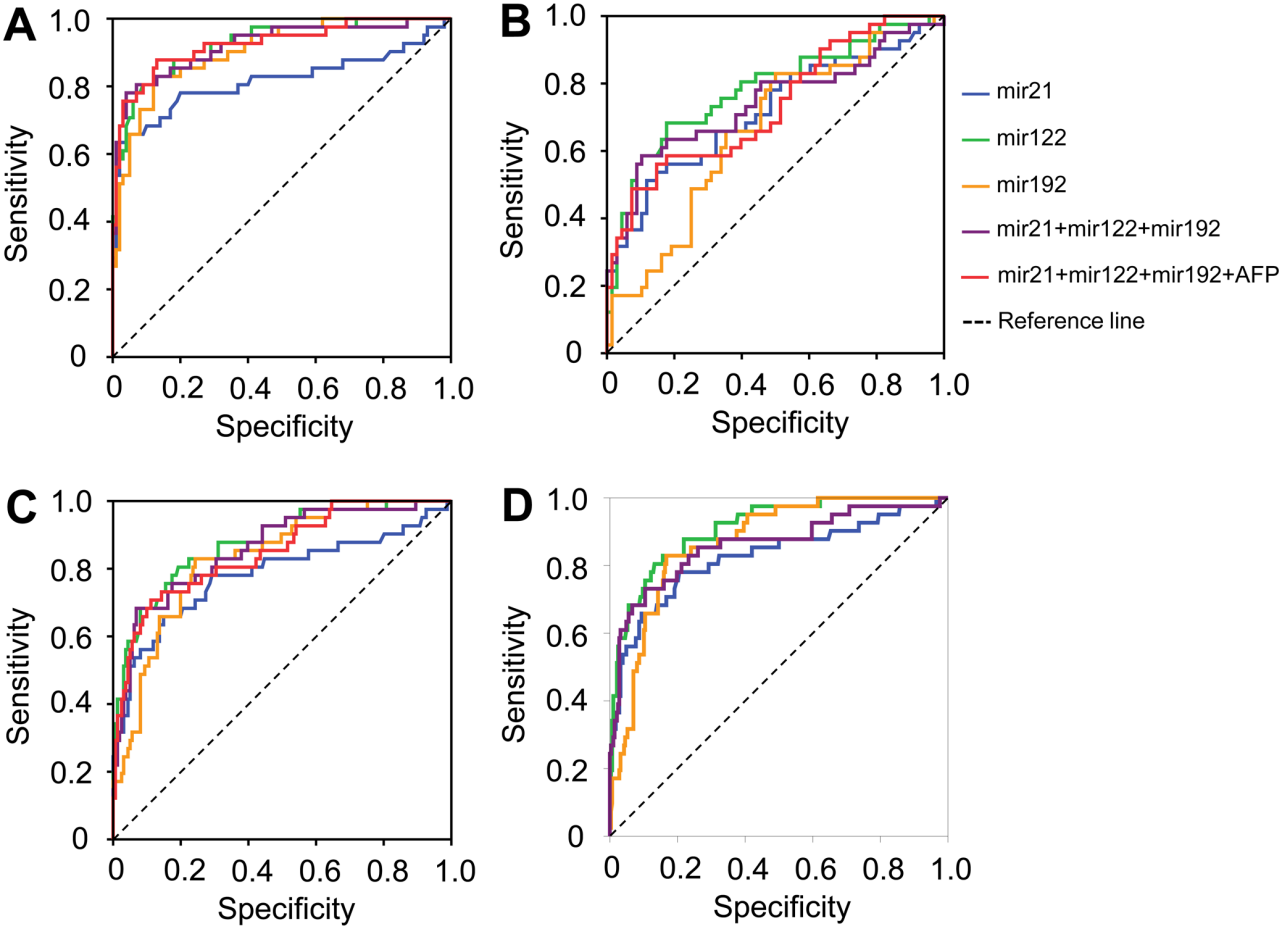
Diagnostics performance of triplex miRNA panel in patients with normal AFP level. Diagnostic performance of the panel Mir@AFP based on the miRNAs miR-21, miR-122, miR-192 and AFP in differentiating hepatocellular carcinoma with normal AFP levels from other conditions (<20 ng/l). (A): HCC vs. CHB; (B): HCC vs. LC; (C): HCC vs. LC+CHB and (D): HCC vs. (LC+CHB+HC).

Individual levels of miR-21, miR-122, miR-192 and AFP showed a moderate diagnostic performance only in differentiating HCC from LC (AUC = 0.698, 0.775, 0.664, and 0.733, respectively) (Table 2 and Fig 3). In contrast, the triplex panel involving the three miRNAs showed in a logistic regression model [triplex_logit(p = HCC) = -1.322 + 0.025miR21 + 0.003miR122 + 0.069miR192] a higher diagnostic accuracy in differentiating HCC from the other conditions. In detail, this applied to CHB patients (AUC = 0.906), patients with either CHB or LC (CHB+LC) (AUC = 0.81) and to non-HCC individuals (CHB+LC+HC) (AUC = 0.854). However, the diagnostic performance to discriminate HCC from LC was slightly lower (AUC = 0.774) (Table 2 and Fig 4). Therefore, we combined the triplex miRNA panel with AFP levels to form the so-called Mir@AFP panel under the logistic regression model [Mir@AFP_logit(p = HCC) = -1.912 + 0.029miR21 + 0.003miR122 + 0.064miR192 + 0.006AFP]. The diagnostic performance in discriminating HCC from other groups provided a higher diagnostic accuracy and reliability (HCC vs. LC, AUC = 0.887; HCC vs. CHB, AUC = 0.948; HCC vs. CHB+LC, AUC = 0.887) if compared to the triplex miRNA panel alone (Table 2 and Fig 3).

**Table 2:** Diagnostic performance of miRNA panels in combination with alpha-fetoprotein in differentiating HCC against control subjects.

Variable(s)	Area Under the Curve (AUC)			
	HCC vs. LC	HCC vs. CHB	HCC vs. (CHB+LC)	HCC vs. (CHB+LC+HC)
miR-21	0.698 (*P*<0.05)	0.805 (*P*<0.05)	0.757 (*P*<0.05)	0.810 (*P*<0.05)
miR-122	0.775 (*P*<0.05)	0.922 (*P*<0.05)	0.862 (*P*<0.05)	0.908 (*P*<0.05)
miR-192	0.664 (*P*<0.05)	0.895 (*P*<0.05)	0.806 (*P*<0.05)	0.872 (*P*<0.05)
AFP	0.733 (*P*<0.05)	0.765 (*P*<0.05)	0.753 (*P*<0.05)	Not applicable
miR21+miR122+miR192 ^(a)^	0.774 (*P*<0.05)	0.906 (*P*<0.05)	0.810 (*P*<0.05)	0.854 (*P*<0.05)
miR21+miR122+miR192 ^(b)^	0.744 (*P*<0.05)	0.922 (*P*<0.05)	0.836 (*P*<0.05)	0.862 (*P*<0.05)
miR21+miR122+miR192+AFP ^(a)^	0.887 (*P*<0.05)	0.948 (*P*<0.05)	0.887 (*P*<0.05)	Not applicable
miR21+miR122+miR192+AFP ^(b)^	0.734 (*P*<0.05)	0.925 (*P*<0.05)	0.850 (*P*<0.05)	Not applicable
miR-21 ^(c)^	0.714 (*P*<0.05)	0.798 (*P*<0.05)	0.764 (*P*<0.05)	0.811 (*P*<0.05)
miR-122 ^(c)^	0.799 (*P*<0.05)	0.933 (*P*<0.05)	0.878 (*P*<0.05)	0.920 (*P*<0.05)
miR-192 ^(c)^	0.692 (*P*<0.05)	0.922 (*P*<0.05)	0.828 (*P*<0.05)	0.888 (*P*<0.05)
AFP ^(c)^	0.742 (*P*<0.05)	0.772 (*P*<0.05)	0.760 (*P*<0.05)	Not applicable
miR21+miR122+miR192 ^(c)^	0.805 (*P*<0.05)	0.912 (*P*<0.05)	0.849 (*P*<0.05)	0.883 (*P*<0.05)
miR21+miR122+miR192+AFP ^(c)^	0.913 (*P*<0.05)	0.967 (*P*<0.05)	0.920 (*P*<0.05)	Not applicable

CHB: chronic hepatitis B; LC: HBV-related liver cirrhosis; HCC: HBV-related hepatocellular carcinoma; AFP: alpha-fetoprotein;

^(a)^: Diagnostic performance of miRNA panel and in combination with AFP levels in all study subjects;

^(b)^: Diagnostic performance of miRNA panel when patients were stratified according to AFP level lower than 20 ng/l.

^(c)^: Diagnostic performance of each miRNA, the miRNA panel and the miRNA panel in combination with AFP levels in differentiating HCC with small tumor size (<5 cm) from other groups.

When AFP levels were <20 ng/ml, the triplex miRNA panel still sustained its diagnostic accuracy in distinguishing HCC from the other groups (CHB patients AUC = 0.922, LC patients AUC = 0.744, patients with either CHB and LC (CHB+LC) AUC = 0.836, non-HCC individuals (CHB+LC+HC) AUC = 0.862). In addition, we also analyzed the diagnostic performance of the Mir@AFP panel in discriminating HCC from other HBV-related liver diseases in patients with normal AFP levels. The diagnostic performance was similar to that of the triplex miRNA panel (HCC vs. CHB, AUC = 0.925; HCC vs. LC, AUC = 0.734; HCC vs. CHB+LC, AUC = 0.850) (Table 2 and Fig 4).

**Fig 5:**
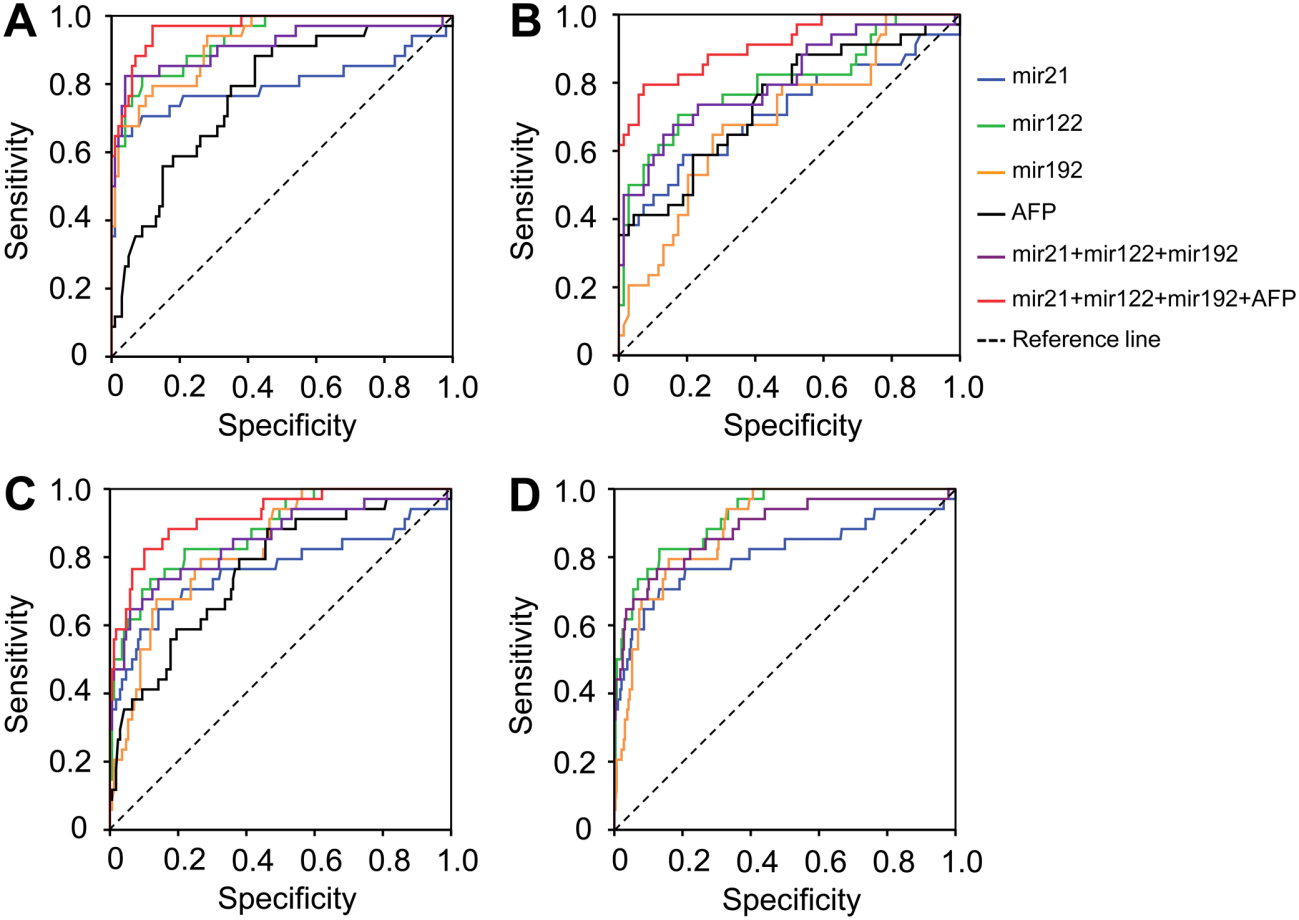
Diagnostics performance of triplex miRNA panel in HCC patients with small tumors. Diagnostic performance of the panel Mir@AFP based on the miRNAs miR-21, miR-122, miR-192 and AFP in differentiating hepatocellular carcinoma with small tumor size (<5 cm) from other groups. (A): HCC vs. CHB; (B): HCC vs. LC; (C): HCC vs. LC+CHB and (D): HCC vs. (LC+CHB+HC).

Out of 118 patients with HBV-related HCC, 35 patients had tumor sizes less than 5 cm. We further analyzed the diagnostic performance of each miRNA, AFP levels, the triplex miRNA and the Mir@AFP in distinguishing HCC patients with small tumor sizes from other HBV patient groups. The triplex miRNA and Mir@AFP panels demonstrated a greater diagnostic performance in distinguishing HCC patients with tumor sizes less than 5 cm from LC (triplex miRNA and Mir@AFP panels, AUC = 0.805 and 0.913, respectively) and from CHB (triplex miRNA and Mir@AFP panels, AUC = 0.912 and 0.967, respectively) (Table 2 and Fig 5).

## Discussion

Early non-invasive diagnostics of HCC is challenged by the lack of reliable biomarkers and the limited validity of imaging methods. Therefore, novel diagnostic methods for detection of early stage HCC need to be developed. Recently, blood circulating miRNAs have been proposed as promising biomarkers for HCC, and the three miRNAs miR-21, miR-122 and miR-192 have already been included in several panels used for HCC screening [13, 15, 16, 25]. However, no single miRNA molecule is accurate enough to reliably support the diagnosis of HCC [26]. In the present study, we evaluated microRNA molecules and established a miRNA panel appropriate for HCC screening. Our results indicate that the combined use of a triplex microRNA panel based on the three miRNAs miR-21, miR-122 and miR-192 and AFP levels significantly improve HCC surveillance in HBV carriers, especially in LC patients with normal AFP levels or HCC patients with small tumor sizes.

So far, several studies have proposed various microRNA panels for HCC screening. However, no consensus in terms of individual microRNA molecules was achieved, even though the studies mentioned were conducted in Chinese ethnics with similar compositions of study groups, including chronic HBV carriers, HBV-related LC and multistage-HCC as well as healthy controls [15, 16]. Additionally, the diagnostic panels described in previous studies require up to seven miRNAs plus synthetic miRNAs like cel-miR-67 or cel-miR-39 as a reference for normalising miRNA levels [15, 16]. This not only complicates laboratory performance but is also difficult to standardise. While use of synthetic miRNAs may provide a better controlling accuracy of sample handling, it may ignore natural degradation of endogenous molecules, as the synthetic miRNAs may acquire better stability than the internal counterparts like miR-16 or U6 [21].

In our study, we re-evaluated expression of miRNA molecules that so far have been suggested as biomarkers for HCC screening [14–16]. Our results revealed that six out of 13 miRNAs (miR-29a, miR-29c, miR-133a, mir-21, mir-122, mir-192) were differentially expressed in the serum of Vietnamese patients with HBV-related HCC compared to CHB patients. Circulating levels of miRNA-21, miRNA-122 and miRNA-192 were correlated with AFP levels and miRNA-122 and miRNA-192 levels were significantly higher in patients with elevated ALT. Six miRNAs (miR-29a, miR-29c, miR-133a, miR-21, miR-122 and miR-192), which showed differential expression in our experiments, were also part of diagnostic panels of previous studies [15, 16]. miR-29a, miR-29c and miR-133a provided only weak RT-PCR fluorescent signals in our experiments. Since weak fluorescence may increase the risk of confusion with arteficial signals we excluded these miRNAs from further analyses.

Consequently, we established a triplex miRNA panel composed of miR-21, miR-122 and miR-192, which showed valid RT-PCR fluorescence signals, and validated its diagnostic performance for HCC surveillance. The triplex miRNA panel demonstrated a higher diagnostic accuracy and reliability compared to the biomarker AFP alone in differentiating individuals with HCC from other groups of patients with HBV-related liver diseases. Our study also shows that the combined use of the triplex miRNA panel based on miRNA -21, miRNA-122 and miRNA-192 and AFP levels can significantly enhance the validity of HCC diagnosis. Therefore, the Mir@AFP panel (miRNA-21, miRNA-122, miRNA-192, and AFP) can be an innovative potent biomarker for screening of HCC in risk populations, thus improving early detection of HCC in HBV patients. Interestingly, our triplex miRNA panel is also able to distinguish HCC patients with normal AFP levels (<20ng/ml) from CHB patients, although it was difficult to discriminate HCC patients, especially HCC patients with AFP levels <20 ng/l, from LC patients. Therefore, we combined the triplex miRNA panel with AFP levels to improve the diagnostic performance. The Mir@AFP panel was rather able to discriminate HCC from other HBV-related liver disease groups, especially from LC patients, compared to either the triplex miRNA panel or AFP alone. Importantly, in the HCC patients with small tumor sizes (<5 cm), our triplex miRNA and Mir@AFP panels also showed a greater diagnostic performance in distinguishing HCC from the other HBV-related liver diseases, especially from patients with LC. These results suggest that the triplex miRNA and Mir@AFP panels can be used for HCC surveillance, particularly in patients with low serum AFP levels and/or patients with low tumor burden.

The ubiquitous oncogenic miR-21 is known to be overrepresented in sera of individuals with various malignancies, including liver cancers. miR-21 levels dynamically change, depending on the clinical presentation; they increase if patients do not respond favorably to chemotherapy [27]. The miRNAs miR-122 and miR-192 are liver specific and their serum levels are elevated in most liver diseases [26, 28]. In addition, miR-21, miR-122 and miR-192 serum levels were associated with different stages of HCC and tumor size, suggesting liver impairment or injury [28]. Of note, miR-192 expression is negatively correlated with HCC metastases, indicating its potential as a marker for HCC patient outcomes [29]. Expression of miR-122 and miR-192 was significantly higher in patients with elevated ALT levels. Although the UAC of miR-122 was superior to that of the triplex miRNA panel, miR-122 could not be used exclusively for the diagnosis of HCC, as over-expression of miR-122 is not specific for HBV-related liver diseases. Previous studies have shown that serum abundance of liver-specific miR-122 is also associated with liver necroinflammation and with liver injuries caused by HCV, alcohol or drugs [30, 31]. Therefore, other miRNAs that are biologically associated with HBV-related HCC (miR-21, miR-192) need to be included to further increase the specificity of HBV-related HCC diagnostics. In addition, miR-122 plays an important role in regulating differentiation, proliferation and maturation of hepatocytes [32, 33]; miR-122 is also involved in HCC tumorgenesis via the transcription process of *c-Myc* [34]. Therefore, the diagnostic capacity of miR-122 does not only indicate the higher number of living differentiated hepatocytes in the liver of HCC patients compared to LC patients, but also indicates abnormal differentiation of hepatocytes in HCC patients. Nevertheless, more studies are needed to clarify the role of miR-122 in tumorgenesis and HCC diagnostic surveillance.

In conclusion, the three miRNAs miR-21, miR-122 and miR-192 were differentially expressed in patients with different stages of HBV-related liver disease progression and correlated with AFP levels. In particular, miR-122 and miR-192 levels were higher in patients with elevated ALT levels. The panel based on these three miRNAs in combination with AFP levels significantly enhances the diagnostic surveillance of HCC in individuals with HBV-related liver diseases, especially in LC patients and HBV patients with normal AFP levels or HCC patients with small tumor sizes.

## Supporting information

S1 Table Primers used for cDNA synthesis and quantitation of microRNAs. (DOC)

S2 Table Ct value and relative expression of miRNAs in the screening phase. (DOC)

S3 Table Serum level of mir-192 was contradictory to Barcelona Clinic Liver Cancer staging. (DOC)

S4 Table The correlation between expression level of miR-21, miR-122, miR192 and study subjects’ age. (DOC)

S1 Fig Differential expression of individual miRNAs used in screening phase. (DOC)

S2 Fig Correlation between circulating levels of microRNAs and AFP levels. (DOC)

S3 Fig Relationship between miRNA and ALT levels. (DOC)

S4 Fig Levels of miRNAs and AFP in subgroups of patients with different tumor sizes. (DOC)

## Abbreviations

AUC: area under the curve
CHB: chronic hepatitis B
HC: healthy controls
HCC: Hepatocellular carcinoma
LC: liver cirrhosis
ROC: receiver operating characteristic
