# Supplementary material for: Optimisation of quantitative miRNA panels to consolidate the diagnostic surveillance of HBV-related hepatocellular carcinoma
Source: PLoS One. 2018 Apr 19;13(4):e0196081. doi: 10.1371/journal.pone.0196081 (PMC5908085; doi:10.1371/journal.pone.0196081)
Supplement: S1 Table — (DOC) [file pone.0196081.s001.doc]

Supplementary Table 1: Primers used for cDNA synthesis and quantitation of microRNAs

| Studied MicroRNA | Mirbase Accession code | Stem-loop primer | Forward primer |
| --- | --- | --- | --- |
| Mir-16 | MIMAT0000069 | gtcgtatccagtgcagggtccgaggtattcgcactggatacgaccgccaa | cgcgctagcagcacgtaaat |
| Mir-21 | MIMAT0000076 | gtcgtatccagtgcagggtccgaggtattcgcactggatacgactcaaca | gcccgctagcttatcagactgatg |
| hsa-miR-26a-5p | MIMAT0000082 | gtcgtatccagtgcagggtccgaggtattcgcactggatacgacagcct | ttcaagtaatccaggatggtcca |
| miR-27a-3p | MIMAT0000084 | gtcgtatccagtgcagggtccgaggtattcgcactggatacgacgcgg | ttcacagtggctaagttggcgca |
| Mir 29a | MIMAT0000086 | gttggctctggtgcagggtccgaggtattcgcaccagagccaac taaccg | tggggtagcaccatctgaaat |
| Mir 29c | MIMAT0000681 | gttggctctggtgcagggtccgaggtattcgcaccagagccaac taaccg | gggtggtagcaccatttgaaat |
| Mir 122 | MIMAT0000421 | gttggctctggtgcagggtccgaggtattcgcaccagagccaac caaaca | ggtgtggagtgtgacaatgg |
| Mir 192 | MIMAT0000222 | gttggctctggtgcagggtccgaggtattcgcaccagagccaac ggctgt | tgtgttggctgacctatgaattg |
| Mir 223 | MIMAT0004570 | gttggctctggtgcagggtccgaggtattcgcaccagagccaac aactca | tggttttggggcgtgtatttgacaa |
| Mir 133a | MIMAT0000427 | gttggctctggtgcagggtccgaggtattcgcaccagagccaac cagctg | tgttttttttttggtccccttcaac |
| Mir 143 3p | MIMAT0000435 | gttggctctggtgcagggtccgaggtattcgcaccagagccaac gagcta | tggtttgtgagatgaagcactg |
| hsa-miR-145-5p | MIMAT0000437 | gtcgtatccagtgcagggtccgaggtattcgcactggatacgacagggat | gtccagttttcccaggagctag |
| hsa-miR-505-5p | MIMAT0004776 | gtcgtatccagtgcagggtccgaggtattcgcactggatacgacacatc | gggagccaggaagtattccta |
| hsa-miR-18b-5p | MIMAT0001412 | gtcgtatccagtgcagggtccgaggtattcgcactggatacgacctaact | taaggtgcatctagtgcactc |
| universal reverse primer | | gtgcagggtccgaggt |  |
