# Supplementary material for: Optimisation of quantitative miRNA panels to consolidate the diagnostic surveillance of HBV-related hepatocellular carcinoma
Source: PLoS One. 2018 Apr 19;13(4):e0196081. doi: 10.1371/journal.pone.0196081 (PMC5908085; doi:10.1371/journal.pone.0196081)
Supplement: S2 Table — (DOC) [file pone.0196081.s002.doc]

**Supplementary Table 2: Ct value and relative expression of miRNAs in the screening phase**

| **miRNAs** | **mir-21** | **mir-29a** | **mir-29c** | **mir-122** | **mir-133a** | **mir-192** | **mir-223 t** | **mir-143 3p** |
| --- | --- | --- | --- | --- | --- | --- | --- | --- |
| Average Ct value | 27.4 ± 2.1 | 36.8 ± 4.5 | 39. ± 5.2 | 26.7± 1.6 | 36.5± 3.5 | 27. ± 2.0 | 36.5± 2.5 | 41.5± 3.2 |
| HCC mir-16 Normalized average expression | 43.149 | 0.160 | 0.079 | 439.487 | 0.393 | 0.429 | 0.003 | 2.617 |
| CHB mir-16 Normalized average expression | 5.406 | 0.009 | 0.002 | 28.917 | 0.034 | 0.042 | 0.001 | 1.266 |
| Rounded average change ratios | 8.0 | 18.0 | 46.0 | 15.0 | 11.0 | 10.0 | 2.0 | 2.0 |
