# Supplementary material for: Optimisation of quantitative miRNA panels to consolidate the diagnostic surveillance of HBV-related hepatocellular carcinoma
Source: PLoS One. 2018 Apr 19;13(4):e0196081. doi: 10.1371/journal.pone.0196081 (PMC5908085; doi:10.1371/journal.pone.0196081)
Supplement: S3 Table — (DOC) [file pone.0196081.s003.doc]

**Supplementary Table 3: Serum level of mir-192 was contradictory to Barcelona Clinic Liver Cancer staging**

| **miRNAs** | **BCLC staging** | **Medium** | **SD** | ***P* value** |
| --- | --- | --- | --- | --- |
| mir-21 | A (n=5) | 110.63 | 207.53 | *P*_(B,C)_ = 0.559 |
|  | B (n=67) | 268.35 | 772.78 |  |
|  | C (n=36) | 85.95 | 211.67 |  |
| mir-122 | A (n=5) | 3424.6 | 4844.11 | *P*_(B,C)_ = 0.078 |
|  | B (n=67) | 4240.1 | 11351.69 |  |
|  | C (n=36) | 1478.26 | 3593.77 |  |
| mir-192 | A (n=5) | 58.62 | 126.04 | ***P*_(B,C)_ < 0.05** |
|  | B (n=67) | 13.35 | 34.41 |  |
|  | C (n=36) | 6.78 | 18.06 |  |
| AFP | A (n=5) | 342.55 | 736.51 | *P*_(B,C)_ = 0.731 |
|  | B (n=67) | 576.98 | 2087.82 |  |
|  | C (n=36) | 370.09 | 604.86 |  |

BCLC: Barcelona Clinic Liver Cancer; SD: Standard deviation;
