# Supplementary material for: Optimisation of quantitative miRNA panels to consolidate the diagnostic surveillance of HBV-related hepatocellular carcinoma
Source: PLoS One. 2018 Apr 19;13(4):e0196081. doi: 10.1371/journal.pone.0196081 (PMC5908085; doi:10.1371/journal.pone.0196081)
Supplement: S4 Table — (DOC) [file pone.0196081.s004.doc]

**Supplementary Table 4: The correlation between expression level of miR-21, miR-122, miR192 and study subjects’** age.

| Age | Speaman’s rho | | | | p |
| --- | --- | --- | --- | --- | --- |
|  | **HCC** | **LC** | **CHB** | **HC** |  |
| miR-21 | 0.179 | 0.15 | 0.058 | 0.044 | > 0.05 |
| miR-122 | 0.148 | 0.016 | -0.106 | -0.12 |  |
| miR-192 | 0.027 | 0.099 | -0.118 | -0.093 |  |
