# Supplementary material for: Optimisation of quantitative miRNA panels to consolidate the diagnostic surveillance of HBV-related hepatocellular carcinoma
Source: PLoS One. 2018 Apr 19;13(4):e0196081. doi: 10.1371/journal.pone.0196081 (PMC5908085; doi:10.1371/journal.pone.0196081)
Supplement: S1 Fig — (DOC) [file pone.0196081.s005.doc]

**Supplementary figure 1: Differential expression of individual miRNAs used in screening phase:** The relative expression of the eight studied miRNAs were compared between eight HCC patients and eight CHB patients. The change ratios between compared groups for each miRNA are presented.
