# Supplementary material for: Optimisation of quantitative miRNA panels to consolidate the diagnostic surveillance of HBV-related hepatocellular carcinoma
Source: PLoS One. 2018 Apr 19;13(4):e0196081. doi: 10.1371/journal.pone.0196081 (PMC5908085; doi:10.1371/journal.pone.0196081)
Supplement: S2 Fig — (DOC) [file pone.0196081.s006.doc]

**Supplementary figure 2: Correlation between circulating levels of microRNAs and AFP levels:** The correlations of microRNAs circulating levels miR-21 (A), miR-122 (B) and miR-192 (C) with alpha-fetoprotein levels were analysed by Spearman's rank correlation coefficient. Spearman's rho and corresponding *P* values are presented.
