# Supplementary material for: Optimisation of quantitative miRNA panels to consolidate the diagnostic surveillance of HBV-related hepatocellular carcinoma
Source: PLoS One. 2018 Apr 19;13(4):e0196081. doi: 10.1371/journal.pone.0196081 (PMC5908085; doi:10.1371/journal.pone.0196081)
Supplement: S3 Fig — (DOC) [file pone.0196081.s007.doc]

**Supplementary figure 3: Relationship between miRNA and ALT levels**

CHB patients were classified into two groups based on the ALT levels (≤40 and >40 IU/ml), and the relative expressions of miR-192 (A) and miR-122 (B) were compared between these classified CHB subgroups. *P* values given were calculated by non-parametric Mann-Whitney U-test.
