# Supplementary material for: Optimisation of quantitative miRNA panels to consolidate the diagnostic surveillance of HBV-related hepatocellular carcinoma
Source: PLoS One. 2018 Apr 19;13(4):e0196081. doi: 10.1371/journal.pone.0196081 (PMC5908085; doi:10.1371/journal.pone.0196081)
Supplement: S4 Fig — (DOC) [file pone.0196081.s008.doc]

**Supplementary figure 4: Levels of miRNAs and AFP in subgroups of patients with different tumor sizes**

HCC patients were classified into subgroups based on the tumor size (smaller than 3 cm, from 3 to <5 cm, from 5 to <10 cm, and great than 10 cm). The relative expressions of miR-21, miR-122 and miR-192 were compared among these classified HCC subgroups. *P* values were calculated by non-parametric Kruskal-Wallis test.
